# Acute and long-term exercise adaptation of adipose tissue and skeletal muscle in humans: a matched transcriptomics approach after 8-week training-intervention

**DOI:** 10.1038/s41366-023-01271-y

**Published:** 2023-02-11

**Authors:** Simon I. Dreher, Martin Irmler, Olga Pivovarova-Ramich, Katharina Kessler, Karsten Jürchott, Carsten Sticht, Louise Fritsche, Patrick Schneeweiss, Jürgen Machann, Andreas F. H. Pfeiffer, Martin Hrabě de Angelis, Johannes Beckers, Andreas L. Birkenfeld, Andreas Peter, Andreas M. Niess, Cora Weigert, Anja Moller

**Affiliations:** 1 Institute for Clinical Chemistry and Pathobiochemistry, Department for Diagnostic Laboratory Medicine, University Hospital Tübingen, 72076 Tübingen, Germany; 2 Institute of Experimental Genetics, Helmholtz Zentrum München, Ingolstädter Landstr. 1, 85764 Neuherberg, Germany; 3 German Center for Diabetes Research (DZD), 85784 Neuherberg, Germany; 4 Department of Clinical Nutrition, German Institute of Human Nutrition Potsdam-Rehbruecke (DIfE), 14558 Nuthetal, Potsdam Germany; 5 Research Group Molecular Nutritional Medicine, German Institute of Human Nutrition Potsdam-Rehbruecke, 14558 Nuthetal, Germany; 6 Charité-Universitätsmedizin Berlin, corporate member of Freie Universität Berlin, Humboldt-Universität zu Berlin, and Berlin Institute of Health, Department of Endocrinology, Diabetes and Nutrition, Campus Benjamin Franklin, 12203 Berlin, Germany; 7 Berlin-Brandenburg Center for Regenerative Therapies (BCRT), Charité - Universitätsmedizin Berlin, corporate member of Freie Universität Berlin, Humboldt-Universität zu Berlin, and Berlin Institute of Health (BIH), 13353 Berlin, Germany; 8 Next Generation Sequencing Core Facility, Medical Faculty Mannheim, Heidelberg University, 68167 Mannheim, Germany; 9 Institute for Diabetes Research and Metabolic Diseases of the Helmholtz Zentrum München, University of Tübingen, Tübingen, Germany; 10 Sports Medicine, University Hospital Tübingen, 72076 Tübingen, Germany; 11 Interfaculty Research Institute for Sport and Physical Activity, University of Tübingen, Tübingen, Germany; 12 Section on Experimental Radiology, Department of Diagnostic and Interventional Radiology, University Hospital Tübingen, 72076 Tübingen, Germany; 13 Chair of Experimental Genetics, Technical University of Munich, 85354 Freising, Germany; 14 Department of Internal Medicine IV, University Hospital Tübingen, 72076 Tübingen, Germany

**Keywords:** Type 2 diabetes, Pre-diabetes, Energy metabolism, Translational research, Adipocytes

## Abstract

**Background:**

Exercise exerts many health benefits by directly inducing molecular alterations in physically utilized skeletal muscle. Molecular adaptations of subcutaneous adipose tissue (SCAT) might also contribute to the prevention of metabolic diseases.

**Aim:**

To characterize the response of human SCAT based on changes in transcripts and mitochondrial respiration to acute and repeated bouts of exercise in comparison to skeletal muscle.

**Methods:**

Sedentary participants (27 ± 4 yrs) with overweight or obesity underwent 8-week supervised endurance exercise 3×1h/week at 80% VO2peak. Before, 60 min after the first and last exercise bout and 5 days post intervention, biopsies were taken for transcriptomic analyses and high-resolution respirometry (*n* = 14, 8 female/6 male).

**Results:**

In SCAT, we found 37 acutely regulated transcripts (FC > 1.2, FDR < 10%) after the first exercise bout compared to 394, respectively, in skeletal muscle. Regulation of only 5 transcripts overlapped between tissues highlighting their differential response. Upstream and enrichment analyses revealed reduced transcripts of lipid uptake, storage and lipogenesis directly after exercise in SCAT and point to β-adrenergic regulation as potential major driver. The data also suggest an exercise-induced modulation of the circadian clock in SCAT. Neither term was associated with transcriptomic changes in skeletal muscle. No evidence for beigeing/browning was found in SCAT along with unchanged respiration.

**Conclusions:**

Adipose tissue responds completely distinct from adaptations of skeletal muscle to exercise. The acute and repeated reduction in transcripts of lipid storage and lipogenesis, interconnected with a modulated circadian rhythm, can counteract metabolic syndrome progression toward diabetes.

## New & noteworthy

Transcriptional alterations demonstrate that adipose tissue responded to exercise completely distinct from skeletal muscle in the same human subjects. Adipose tissue showed acute and repeated reduction in transcripts of lipid uptake, synthesis and storage. The data also suggest the restoration of a healthier circadian rhythm in adipose tissue of subjects with obesity after regularly performed exercise. The interconnection of circadian rhythm with lipid metabolism possibly contributes to improved metabolic health and prevention of diabetes.

## Graphical abstract

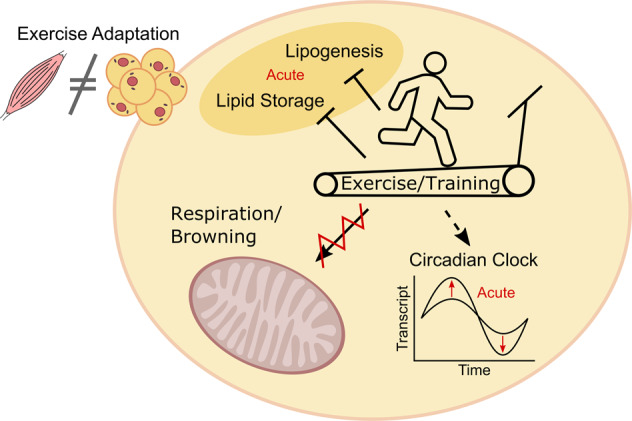

## Introduction

The onset of type 2 diabetes (T2D) is mostly driven by lifestyle and other environmental factors [1]. Tipping the balance between the two main factors that influence the risk for T2D development, nutrition and physical activity, causes weight gain with increased body fat and reduced cardiorespiratory fitness as consequences of a hypercaloric nutrition in combination with a sedentary lifestyle [2]. Regular physical activity can prevent or revert this state even without dietary restrictions, markedly reducing the risk for T2D [3–5]. Physical exercise not only preserves insulin sensitivity in healthy persons but can restore compromised insulin sensitivity in most subjects with overweight or prediabetes [6].

As the predominantly utilized organ, skeletal muscle (SM) and its relevance for beneficial effects of physical activity have been extensively characterized [7–10]. Adaptations involved in this metabolic benefit are increased SM mass, capillarization, and mitochondrial content thereby supporting SM glucose disposal in response to insulin [7, 11–13]. Besides the SM, other tissues also contribute to metabolic homeostasis. Fat storage in the subcutaneous adipose tissue (SCAT) represents the normal physiological buffer for an imbalance in excess energy intake and limited energy expenditure but is also linked to development of insulin resistance and T2D [14–17]. In healthy human subjects, long-term endurance training was reported to improve SCAT insulin sensitivity [18, 19]. Comparison of endurance-trained and untrained humans revealed higher abundance of enzymes of lipolysis, glyceroneogenesis and oxidative phosphorylation in SCAT of the trained subjects [20]. In addition, data on SCAT adaptations after long-term training interventions centered around results on the potential adaptation of mitochondrial respiration and content [19, 21–26]. Thus, long-term adaptations of SCAT to training might alter mitochondrial respiration and affect lipid metabolism in SCAT.

The contracting SM undergoes dramatic transcriptomic and (phospho)proteomic changes in response to acute exercise, which trigger the functional adaptative processes [13, 27]. Less is known about the transcriptomic response of SCAT to one and repeated bouts of exercise. We hypothesize, that physically not directly utilized tissues like SCAT also acutely respond to one bout of exercise which paves the way for long-term effects on lipid metabolism and potentially mitochondrial function.

Thus, in this study, we aimed to characterize SCAT transcript levels in response to acute and repeated bouts of exercise in comparison to changes in SM of matched human donors. Untrained subjects with overweight and obesity performed an 8-week supervised endurance training intervention. After the 8 weeks, participants had improved cardiorespiratory fitness and individual lactate threshold and reduced adiposity, also detected by magnetic resonance imaging as decrease in total and SCAT volume [26]. To elucidate whether the observed reduction in SCAT volume is accompanied and potentially triggered by molecular responses in this tissue to exercise, we used an unbiased exploratory approach, analyzed and compared transcriptomic changes after one acute bout of exercise in the untrained and trained state as well as long-term training adaptations in abdominal SCAT biopsies and vastus lateralis muscle biopsies from the same subjects.

## Methods

### Study participants

Healthy, sedentary (<120 min of physical activity per week) humans at high risk for T2D with at least one of the following risk factors (BMI > 27 kg/m2, family history (first degree) of T2D, former gestational diabetes) were recruited. Out of 25 subjects that were included in the previous data analysis [26] a total of 14 subjects were included in the present study due to criteria detailed below. All participants gave written informed consent and the study protocol was approved by the ethics committee of the University of Tübingen and was in accordance with the declaration of Helsinki. The study was registered at Clinicaltrials.gov as trial number NCT03151590.

### Study design

Participants performed 1 h of supervised endurance training three times per week for 8 weeks, consisting of 30 min of cycling and 30 min of walking on a treadmill. Before and after the training period, all participants underwent maximal spiroergometry as an incremental cycling test using an electromagnetically braked bicycle ergometer, to determine the individual VO2peak. The test was terminated at volitional exhaustion or muscular fatigue. Peak VO2 was defined as the mean VO2 over the last 20 s before the cessation of exercise and was assessed by metabolic gas analysis. The training intensity was individually set at 80% of the VO2peak determined before the intervention and was not changed throughout the training period. Training intensity was controlled by heart rate based on predetermined 80% of the VO2peak and individually set. For anthropometric data of the included 14 participants, see Tab. S1. Body fat mass and distribution were measured by magnetic resonance imaging [28]. SCAT volume was analyzed from the femoral heads to humeri. Metabolic and fitness parameters were assessed as described in [26].

### Biopsy collection

Subcutaneous adipose tissue biopsies were performed under sterile conditions after local anesthesia (2% Scandicaine, Astra Zeneca, Wedel, Germany) going back and forth lateral of the umbilicus with a Menghini-needle (Hepafix, B. Braun, Melsungen, Germany). Skeletal muscle biopsies were obtained from the lateral part of vastus lateralis muscle. After local anesthesia, skin, fat tissue, fascia, and the muscle epimysium were cut under sterile conditions using a scalpel, and a piece of muscle was removed using a Bergström needle (Pelomi Medical, Albertslund, Denmark) with suction [26]. Biopsies were taken 8 days before (Baseline) and 5 days after (Trained) the 8-week intervention in a resting state 60 min after the end of an OGTT as well as 60 min after the first (Untrained Acute) and last 30 min ergometer exercise bout (Trained Acute). 45 min before the acute exercise bout, participants received a defined breakfast to account for the glucose-induced hormonal changes after the OGTT in the rested state biopsies (Fig. S1) All biopsies were collected at 11:00 am ± 30 min.

### Transcriptomic analyses

Total RNA was isolated from snap-frozen adipose tissue using Trifast-Chloroform extraction (Peqlab) with the miRNeasy Kit (Qiagen) and skeletal muscle biopsies with the miRNeasy Kit, each including DNAse digestion. Both tissues were homogenized using a TissueLyser II (Qiagen). Only high-quality RNA (RNA integrity number >7, Agilent 2100 Bioanalyzer) was used for microarray analysis. Total RNA was amplified using the WT PLUS Reagent Kit (Thermo Fisher Scientific Inc., Waltham, USA). Amplified cDNA was hybridized on Human Clariom S arrays (Thermo Fisher Scientific). Staining and scanning (GeneChip Scanner 3000 7 G) was done according to manufacturer’s instructions. From a total of 14 out of the 25 participants, transcriptome data of both adipose tissue and skeletal muscle were available resulting in *n* = 11 samples at the first resting timepoint (Baseline), *n* = 10 after the first (Untrained Acute), *n* = 13 after the last (Trained Acute) acute bout of exercise and *n* = 12 at the resting timepoint after the training (Trained). A total of *n* = 6 participants were sampled at all timepoints. For evaluation of transcriptional changes (FC) in matched samples of subjects (adipose tissue and skeletal muscle) at respective timepoints, samples of *n* = 8 acute untrained and *n* = 9 for training effects could be compared (Fig. S1). Transcriptome Analysis Console (TAC; version 4.0.0.25; Thermo Fisher)) was used for quality control and to obtain annotated normalized SST-RMA gene-level data. Statistical analyses were performed with R3.6.3/Rstudio [29]. Array data were submitted to the GEO database at NCBI (GSE208032, GSE161750, GSE161749).

### High-resolution respirometry

Respirometry measurements were performed using Oroboros Oxygraph 2k (Oroboros Instruments GmbH, Innsbruck, Austria) as described previously in [26]. Data of the subset (*n* = 14) of all participants were included in this study. Details were included as Supplementary Information.

### Adipose tissue transcriptomic analysis in the CLOCK study

Transcriptomic analysis implied a close connection between lipid metabolism and circadian rhythm. Therefore, our transcript data was integrated with data from the CLOCK study (NCT02487576). From 15 out of 29 men (age 45.9 ± 2.5 years, BMI 27.1 ± 0.8 kg/m2), transcriptomic analysis of SCAT samples was conducted [30]. SCAT biopsies were collected three times during the investigation day (at 8:40 am, 12:20 pm and 7:00 pm) at the level of the umbilicus by the needle aspiration. Parameters of circadian rhythms of gene expression were estimated by a three-time-point rhythm prediction method [31] using a magnitude correction. Details were included as Supplementary Information.

### Statistical analyses

Statistical analyses were included as Supplementary Information.

## Results

### Acute exercise response and training adaptation of adipose tissue transcriptome

**Fig. 1:**
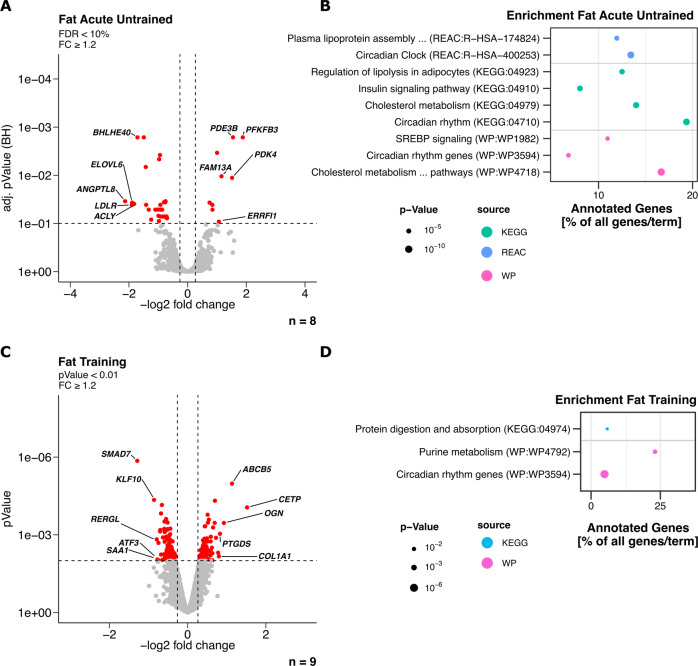
Acute transcriptomic exercise response in subcutaneous adipose tissue (Fat) biopsies of participants that underwent an 8-week training intervention program. Transcriptomic changes were calculated to assess the acute exercise effects (*n* = 8) and the long-term training (*n* = 9) effects as compared to the baseline untrained state. **A** Volcano plot depicting up- and down-regulated transcripts in fat after acute exercise in an untrained state. Transcripts with FC ≥ 1.2 and limma *t*-test with BH correction at FDR < 10% were considered significantly regulated (red). Top 5 regulated transcripts based on FC were labeled. **B** Enrichment analysis of significantly regulated transcripts in fat after acute exercise in an untrained state (FC ≥ 1.2 and limma *t*-test *p* < 0.01). **C** Volcano plot depicting up- and down-regulated transcripts in fat after 8 weeks of training in a rested state FC ≥ 1.2 and limma *t*-test *p* < 0.01. **D** Enrichment analysis of significantly regulated transcripts in fat after training (FC ≥ 1.2 and limma *t-*test *p* < 0.01). Shown are significantly enriched terms.

To identify the initial response of SCAT to an acute bout of exercise in our otherwise untrained participants we analyzed transcriptomic changes occurring after the first bout of exercise (Fig. S1). The untrained-acute response of SCAT involved differential regulation of 37 transcripts with a fold change (FC) ≥ 1.2 (limma *t*-test and BH correction at FDR < 10%). Of these 37 significantly regulated transcripts, the majority (28) were down-regulated with only 9 up-regulated transcripts (Fig. 1A). The significantly regulated transcripts after acute exercise were pronouncedly enriched in pathways associated with lipogenesis, lipid metabolism, and related transcriptional regulators such as PPAR and SREBP (Fig. 1B). Regulated transcripts were also enriched in terms like circadian clock and circadian rhythm in the acute exercise response of SCAT (Fig. 1B). We also assessed the adaptation of SCAT on the transcriptomic level after 8 weeks of training by comparing the baseline biopsies before intervention to the biopsies collected 5 days post intervention. When we applied the same strict statistical criteria only *SMAD7* was significantly lower expressed compared to SCAT before the training intervention. When we eased the statistical threshold to *p* < 0.01 (limma *t*-test) we found 209 differentially expressed transcripts with 115 down- and 94 up-regulated transcripts (Fig. 1C). The changes in the transcriptome were again found to be enriched in the term circadian rhythm genes, whereas terms associated with lipid metabolism were not found (Fig. 1D). Taken together, not directly utilized SCAT responded to exercise with acute transcriptomic changes associated to lipid metabolism and circadian rhythm and showed long-term transcriptomic adaptation to an 8-week training intervention that seemed also associated to the circadian rhythm.

### Regulation of lipid storage and lipogenesis and upstream regulator analysis

**Fig. 2:**
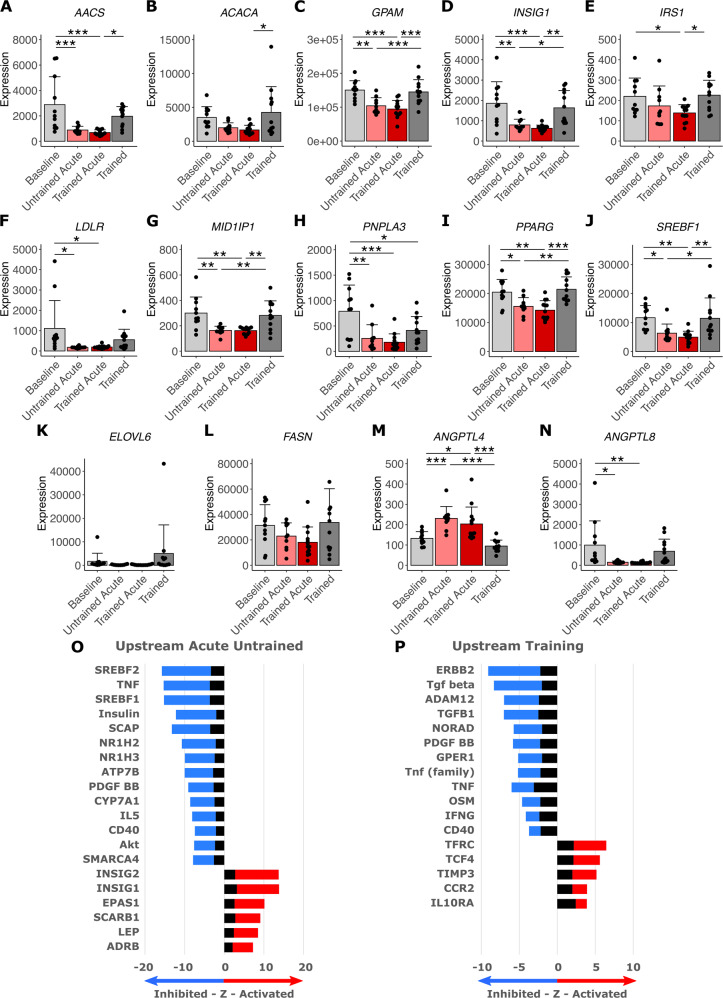
Regulation of genes related to lipid storage and lipogenesis and analysis of predicted upstream regulators. Subcutaneous adipose tissue biopsies of participants that underwent an 8-week training intervention program were analyzed. Biopsies were taken before (Baseline *n* = 11) and after the intervention (Trained *n* = 12) as well as 60 min after the first (Untrained Acute *n* = 10) and last acute exercise bout (Trained Acute *n* = 13). Transcript level of (**A**) *AACS*, (**B**) *ACACA*, (**C**) *GPAM*, (**D**) *INSIG1*, (**E**) *IRS1*, (**F**) *LDLR*, (**G**) *MID1IP1*, (**H**) *PNPLA3*, (**I**) *PPARG*, (**J**) *SREBF1*, (**K**) *ELOVL6*, (**L**) *FASN*, (**M**) *ANGPTL4*, (**N**) *ANGPTL8* was compared between each timepoint. Bars represent mean ± SD, individual data points are depicted. Significant differences were assessed using one-way ANOVA with Tukey correction, *p < 0.05, **p < 0.01, ***p < 0.001, *n* = 10–13 with *n* = 6 represented in all timepoints. Ingenuity Pathway Analysis software was used to predict the activation (*z*-score > 2) or inhibition (*z*-score < −2) of upstream regulators based on transcriptomic changes (FC ≥ 1.2 and limma *t*-test *p* < 0.01) after (**O**) acute untrained exercise (*n* = 8) and (**P**) long-term training (*n* = 9). Stacked bars represent activation *z*-score and −log10p values. Direction is based on a positive or negative *z*-sore indicating activated (red) or inhibited (blue) signaling of upstream regulators. Top 20 upstream regulators based on significant *p* values (*p* < 0.05) were plotted.

As the most enriched pathways after acute exercise were associated with lipid metabolism, we next took a closer look on the gene expression of enzymes associated with lipogenesis and lipid storage (Fig. 2A–N). Expression of most enzymes and proteins relevant for lipogenesis and lipid storage like *AACS*, *ACACA*, *GPAM*, *INSIG1*, *IRS1*, *LDLR*, *MID1IP1*, *PNPLA3*, *PPARG*, and *SREBF1* was significantly lower directly after acute exercise compared to samples taken in the resting state (Fig. 2A–J). Due to inter-subject variation expression of *ELOVL6* and *FASN* did not reach significance after Tukey correction despite showing the same trend (Fig. 2K, L). Significantly elevated after acute exercise was the expression of lipoprotein lipase (LPL) inhibitor *ANGPTL4* while expression of its suppressor *ANGPTL8* was reduced (Fig. 2M, N). The 8 weeks of training had almost no influence on the expression levels after acute exercise or in the resting state. Ingenuity upstream-regulator analysis performed on the transcriptomic data further painted the picture of reduced lipogenesis as acute response of SCAT to exercise (Fig. 2O). Negative z-scores indicate the inhibition of upstream-regulators SREBF1/2, Insulin and AKT while feedback repressors of SREBFs, like INSIG1/2, are activated (Fig. 2O). This feedback loop appears to be actively at play as *INSIG1* expression itself is lower 60 min after acute exercise (Fig. 2D) while overall transcriptomic changes as well as *SREBF1* expression (Fig. 2J) hint toward activated INSIGs and inhibited SREBFs (Fig. 2O). Activated beta-adrenergic receptor (ADRB)-signaling is well in line with exercise-induced catecholamines [32] as potential mediators of exercise responses in SCAT (Fig. 2O). Upstream-regulator analysis of the training response in resting conditions suggests long-term anti-inflammatory adaptation of SCAT with inhibited TGF-β, PDGF, TNF, IFNG and activated anti-inflammatory IL10RA-signaling (Fig. 2P). Upstream analysis also hints toward an acute anti-inflammatory response with inhibited TNF- and PDGF-signaling (Fig. 2O). Taken together, our data show that SCAT acutely responds to exercise with down-regulation of transcripts related to lipogenesis and lipid storage and adapts long-term in an anti-inflammatory manner. Further, the down-regulation of insulin signaling and many insulin-dependent transcripts suggest the activation of counterregulatory hormonal pathways, likely β-adrenergic regulation as major driver of the acute transcriptional response of SCAT to exercise.

### Circadian rhythm during acute exercise and training

Other pathways strikingly associated with the transcriptomic responses of SCAT to acute exercise as well as adaptation to training were related to the circadian rhythm. Core clock genes *CLOCK* and *ARNTL/BMAL1* (Fig. S2A, B) were not significantly altered by acute exercise or training at our sampling time at 11:00 am ± 30 min. On the contrary, the repressors of the core clock genes *PER1*, *PER2*, *CRY2* and *NR1D1* showed all higher expression levels after acute exercise compared to resting timepoints (Fig. S2C–F). Other circadian genes like *BHLHE40*, *BHLHE41* and *RAI1* were lower and *CIART* was higher expressed after acute exercise (Fig. S2G–J). The expression of *BHLHE40/41* was also reduced in the resting state after the 8-week training (Fig. S2G,H).

**Fig. 3:**
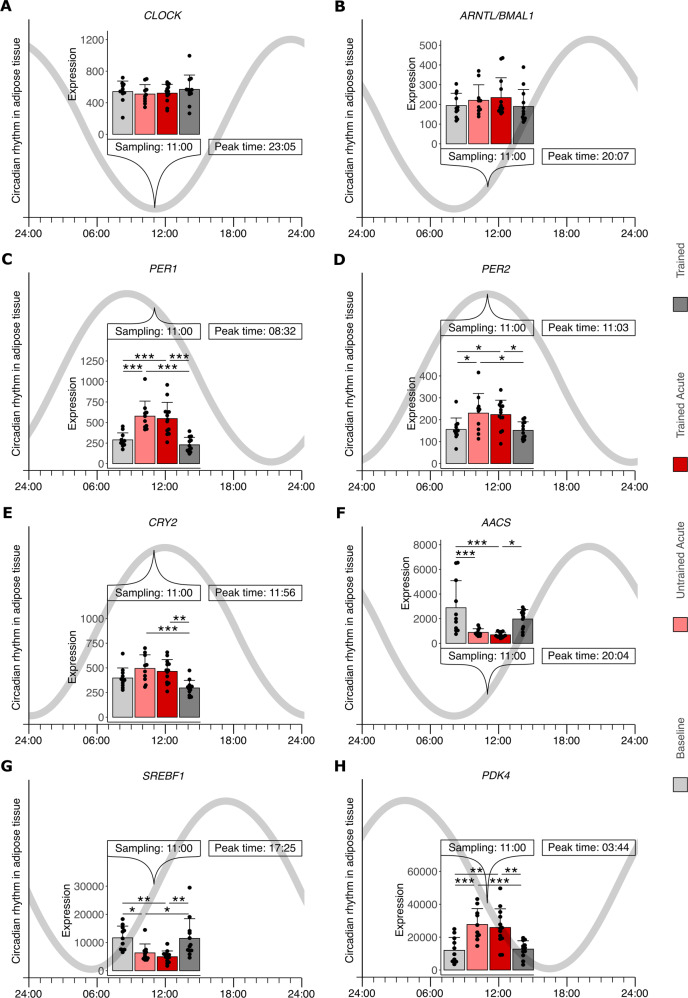
Circadian rhythm in adipose tissue. Subcutaneous adipose tissue biopsies of participants that underwent an 8-week training intervention program were analyzed. All biopsies were taken at 11:00 am ± 30 min, before (Baseline, light gray *n* = 11) and after the intervention (Trained, dark gray *n* = 12) as well as 60 min after the first (Untrained Acute, light red *n* = 10) and last acute exercise bout (Trained Acute, dark red *n* = 13). Transcript levels of (**A**) *CLOCK*, (**B**) *ARNTL/BMAL1*, (**C**) *PER1*, (**D**) *PER2*, (**E**) *CRY2*, (**F**) *AACS*, (**G**) *SREBF1*, (**H**) *PDK4* were integrated with the extrapolated circadian expression [31] of the respective genes in resting humans from samples assessed in [30] and shown in Table S2. The gray line represents extrapolated circadian expression pattern based on peak time data (*y*-axis depicts time of day). Bars represent mean ± SD, individual datapoints are depicted. Significant differences were assessed using one-way ANOVA with Tukey correction, *p < 0.05, **p < 0.01, ***p < 0.001, *n* = 10–13 with *n* = 6 represented in all timepoints.

Our enrichment analysis implied a close connection between lipid metabolism and circadian rhythm (Fig. 1B). Therefore, we next interpreted our transcript data by integrating it with recent experimental data in SCAT of human subjects with similar physiological traits where three samples were taken per day [30] to extrapolate rhythm and peak expression time of each transcript as described previously [31]. This data integration confirmed that many of our significantly regulated transcripts involved in lipid metabolism (Fig. 2) namely *AACS*, *ELOVL6*, *INSIG1*, *IRS1*, *MID1IP1*, *SREBF1* and *PDK4* follow a circadian expression pattern in SCAT (Tab. S2). The extrapolation of the peak expression time of these transcripts in human SCAT allows for evaluation of the exercise or training effects in relation to their current expression status at sampling time (11:00 am ± 30 min) (Fig. 3). The unaffected core clock genes are on their lowest expression during sampling hours (Fig. 3A, B) and repressed by their repressors *PER1*, *PER2*, and *CRY2* that reach their peak expression time in SCAT shortly before, shortly after or right at sampling time in case of *PER2* (Fig. 3C–F). Interestingly, all of these repressors were further elevated during their peak times acutely after exercise (Fig. 3C–E). Other transcripts that were also involved in lipid metabolism like *AACS*, *SREBF1* or *PDK4* were acutely regulated after exercise in a phase of their circadian rhythm in-between lowest and peak expression (Fig. 3F–H). Taken together, integration of our data on regulation by exercise with data on circadian rhythm in SCAT potentially hints toward a change in amplitude or shift of rhythm by acute exercise.

### Acute exercise response and training adaptation in skeletal muscle vs adipose tissue

**Fig. 4:**
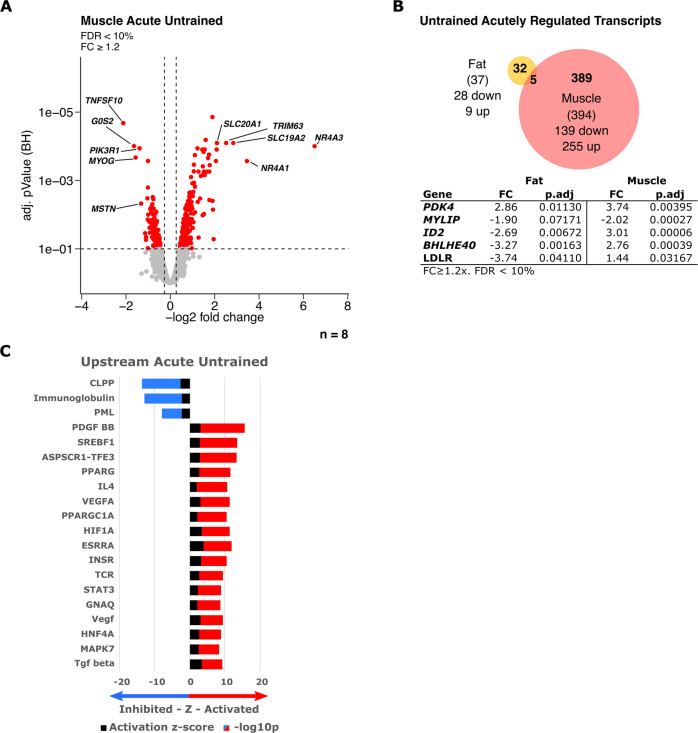
Acute transcriptomic exercise response in skeletal muscle vs adipose tissue. Transcriptomic changes were calculated in matched samples of subjects (Fat and Muscle) to assess the acute exercise effects (*n* = 8). **A** Volcano-Plots depicting up- and down-regulated transcripts in muscle after acute exercise in an untrained state. Transcripts with FC ≥ 1.2 and FDR < 10% were considered significantly regulated (red) and top 5 regulated transcripts based on FC were labeled. **B** Venn-Diagram representing the overlap of significantly regulated transcripts between fat (yellow) and muscle (red) after acute exercise in an untrained state. Significantly regulated transcripts overlapping between fat and muscle are listed in the tables with respective FC and adjusted *p* value (p.adj). **C** Upstream analysis based on transcriptomic changes in muscle (FC ≥ 1.2 and *t*-test *p* < 0.01) to identify significantly altered upstream regulator signaling (*z*-score > 2, *p* < 0.05) as untrained acute response. Stacked bars represent activation *z*-score and −log10p values. Direction is based on a positive or negative *z*-sore indicating activated (red) or inhibited (blue) signaling of upstream regulators. Top 20 upstream regulators based on *p* value are plotted.

SM as the primary utilized organ acutely and extensively adapts to exercise on a molecular level. This became also evident when analyzing the transcriptomic changes in the SM in our matched donors. The acute response of SM tissue involved differential regulation of 394 transcripts with a FC ≥ 1.2 (FDR < 10%). In contrast to the results in SCAT, the majority (255) of these transcripts were up-regulated with less (139) down-regulated transcripts in the same matched donors (Fig. 4A). In addition to the difference in the acute response between SCAT and SM regarding number of regulated transcripts and direction of the majority of regulated transcripts, the most striking observation was the lack of overlap. Only 5 transcripts in the acute response (Fig. 4B) were regulated in both tissues with only 2 regulated in the same direction. Of note, *PDK4* was up- and *MYLIP* down-regulated acutely after exercise (Fig. 4B). The upstream-regulator analysis further underlined the completely distinct adaptation to acute exercise in muscle compared with SCAT (Fig. 4C). While acute adaptation in SCAT involves inhibition of many upstream-regulators affecting lipid metabolism and insulin signaling (SREBFs, SCAP, Insulin, Akt) (Fig. 2O), in SM, most upstream-regulator signaling is activated in response to acute exercise including SREBF1 and PPARG, but also PGC1α (PPARGC1A), VEGF, and HIF1A signaling all of which are drivers of the improved substrate storage and oxidation in the trained SM (Fig. 4C).

**Fig. 5:**
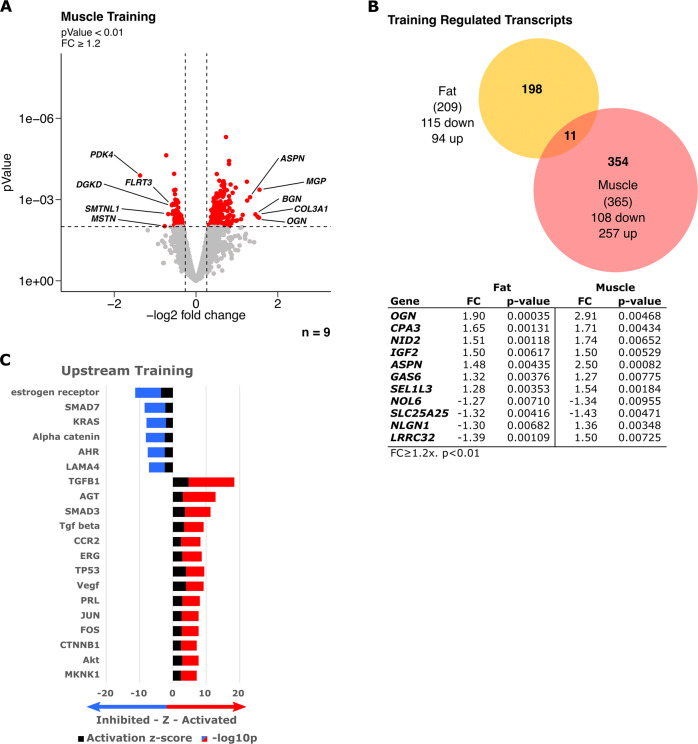
Training adaptation of skeletal muscle vs adipose tissue transcriptome. Skeletal muscle (Muscle) and subcutaneous adipose tissue (Fat) biopsies of participants that underwent an 8-week training intervention program were analyzed. Transcriptomic changes were calculated in matched samples of subjects (fat and muscle) to assess the long-term training (*n* = 9) effects. **A** Volcano plot depicting up- and down-regulated transcripts in muscle after 8 weeks of training in a rested state with FC ≥ 1.2 and limma *t-*test *p* < 0.01. Significantly regulated transcripts are depicted in red and top 5 based on FC were labeled. **B** Venn-Diagram representing the overlap of significantly regulated transcripts between fat (yellow) and muscle (red) after training. Significantly regulated and overlapping transcripts are listed in the table with respective FC and adjusted *p* value (p.adj). **C** Upstream regulator analysis based on transcriptomic changes in muscle (FC ≥ 1.2 and *p* < 0.01) to identify significantly altered upstream regulator activity (*z*-score > 2 or < −2, *p* < 0.05) as long-term training response. Stacked bars represent activation *z*-score and −log10p values. Direction is based on a positive or negative *z*-sore indicating activated (red) or inhibited (blue) signaling of upstream regulators. Top 20 upstream regulators based on *p* value are plotted.

When we analyzed the adaptation of SM on the transcriptomic level after 8 weeks of training in the resting state, no transcript was differentially regulated at an FDR < 10%. However, with a less stringent statistical threshold (*p* < 0.01) 365 transcripts were differentially expressed in SM after 8 weeks of training in a rested state and unlike in SCAT adaptation, more (257) up- than down-regulated (108) transcripts (Fig. 5A). Similar to the acute response, when comparing SCAT with SM we found surprisingly few transcripts regulated in both tissues. Only 11 genes were regulated in both tissues as an adaptation to 8 weeks of training with 9 being regulated in the same direction (Fig. 5B). Upstream-regulator analysis of SM response to training pointed toward cell growth, hypertrophy and extracellular matrix remodeling as long-term transcriptional adaptations (Fig. 5C), in clear contrast to the anti-inflammatory adaptation in SCAT (Fig. 2P). Taken together, an overlap between SCAT and SM transcriptomic adaptation was basically not existent and upstream analysis revealed distinct and in part opposite responses between SM and SCAT of the same donors in response to acute exercise and training.

### Mitochondrial respiration and beigeing/browning

**Fig. 6:**
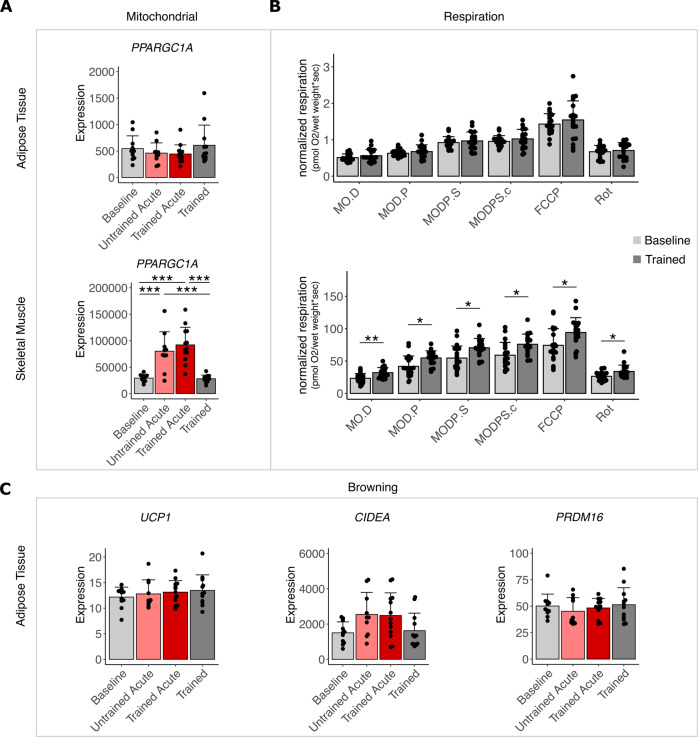
Mitochondrial respiration and beigeing/browning. Skeletal muscle and subcutaneous adipose tissue (biopsies of participants that underwent an 8-week training intervention program were analyzed. Biopsies were taken before (Baseline *n* = 11) and after the intervention (Trained *n* = 12) as well as 60 min after the first (Untrained Acute *n* = 10) and last acute exercise bout (Trained Acute *n* = 13). **A** Transcript level of *PPARGC1A* was compared between each timepoint in fat (top) and muscle (bottom) (*n* = 10–13 with *n* = 6 represented in all timepoints). **B** Respiration of fat (top) and muscle (bottom) was measured in response to indicated substrates before (Baseline) and after (Trained) 8 weeks of training (*n* = 14, results are subset of recently published data [26]). M = malate, O = octanoylcarnitine, D = adenosine diphosphate (ADP), P = pyruvate, S = succinate, c = cytochrome c, FCCP = carbonyl cyanide-p-trifluoromethoxyphenyl-hydrazone Rot = rotenone. **C** Transcript level of *UCP1*, *CIDEA*, *PRDM16* in fat was compared between each timepoint (*n* = 10–13 with *n* = 6 represented in all timepoints). Bars represent mean ± SD, individual datapoints are depicted. Significant differences were assessed using one-way ANOVA with Tukey correction (**A** and **C**) or Fisher’s LSD post hoc test (**B**), *p < 0.05, **p < 0.01, ***p < 0.001.

One of the hallmarks of exercise-adaptations of SM is the increase in mitochondrial respiration. Concordantly, we found a strong and significant induction of the co-transcriptional regulator of mitochondrial maintenance and abundance, *PGC1α* (*PPARGC1A*), acutely after exercise in SM. This was not the case in SCAT where *PGC1α* expression was unchanged (Fig. 6A). This was accompanied by unaltered mitochondrial respiration in SCAT of the biopsy donors, while it was clearly elevated in myofibers obtained from SM after the 8-week training intervention, as also reported recently for the entire study cohort [26] (Fig. 6B). Since beigeing/browning of white SCAT is a consistently reported adaptation to training in animal studies, we also looked at typical markers associated with beigeing/browning or brown adipose tissue in our SCAT biopsies (Fig. 6C). While *UCP1* and *PRDM16* showed unchanged expression, expression of *CIDEA* tended to be acutely elevated which might however also be attributed to its association with the circadian rhythm (Fig. 6C). Thus, only SM but not SCAT adapted to training by increasing its mitochondrial respiration rate and we found no evidence toward beigeing/browning after an 8-week training intervention in our human subjects.

## Discussion

In this study, we utilized an unbiased approach to characterize acute exercise responses and long-term training adaptations of SCAT in comparison to changes in SM of the same human donors. Our data showed that not directly utilized SCAT still acutely responded to exercise with transcriptomic changes and showed long-term adaptation to an 8-week training intervention. Interestingly, an overlap between SCAT and SM transcriptomic changes was basically non-existent suggesting completely distinct forms of adaptation to exercise/training and the regulation thereof. This is further supported by partially opposite regulation of upstream signaling pathways and lack of enhancement in mitochondrial respiration in SCAT.

The data provide clear evidence that SCAT responds to acute exercise with down-regulation of transcripts related to lipogenesis and lipid storage. Gene expression of many proteins and enzymes, relevant for lipid synthesis and storage, were found significantly and robustly down-regulated after acute exercise bouts in both untrained and trained states, extending results of a recent acute exercise study with subjects of obesity [33]. Positive regulators and activators like insulin signaling and SREBF transcription factors, responsible for regulation of many genes needed for uptake and synthesis of fatty acids, cholesterol as well as phospholipids and triglycerides, were reduced. Lipid uptake was also acutely affected by inhibition of LDLR and LPL via regulation of ANGPTLs. In SCAT the most potent LPL inhibitor is ANGPTL4 that can be repressed or sequestered by forming a dimer with ANGPTL8 [34]. In line with reduced lipid uptake and storage, LPL is inhibited by acute up-regulation of *ANGPTL4* and down-regulation of *ANGPTL8*. Lipogenesis was affected in response to acute exercise with reduced acetyl-CoA carboxylase 1 (*ACACA*) representing the first step of fatty acid synthesis. Further acutely reduced transcripts were the key enzyme of fatty acid synthesis *FASN* and *GPAM* catalyzing glycerolipid e.g., triglyceride synthesis and *AACS* that uses ketone bodies for fatty acid and cholesterol synthesis [35, 36]. This clear transcriptional response pattern did not persist as transcriptional long-term adaptation in trained SCAT samples collected 5 days after the last exercise bout. Although we have no data on alterations in protein abundance in the biopsies, it can be speculated that the repeated transcriptional down-regulation of lipid storage and lipogenesis throughout the 8 weeks might contribute to the reduced subcutaneous adipose volume after the training.

One other recent study investigated the response of SCAT to acute exercise in lean participants before and after 6-weeks of training [37]. They reported differentially expressed genes related to inflammation after acute exercise and unlike in our study portrayed a proinflammatory response suggesting exercise-induced macrophage infiltration, in combination with activation of proinflammatory genes in adipocytes. Upstream analysis of our data clearly hinted toward an anti-inflammatory response, however, more pronounced as a long-term adaptation than acute response. These differences might in part be caused by different phenotypes, lean vs obese, of individuals that were recruited in the two studies or study designs where in our 8-week intervention, long-term adaptations had 2 weeks longer to establish.

Recently, an interaction between circadian control of SCAT metabolism and exercise was propagated [38, 39]. We found in our study that particularly the response of SCAT to acute exercise is linked to its circadian rhythm. Cultured mammalian adipocytes were previously described to display circadian rhythms of core clock [40], insulin sensitivity [41], glucose uptake [42] and lipolysis [43] which is in line with findings for SCAT in vivo in mice [44]. Individuals with obesity and T2D showed an overall reduced amplitude of circadian gene expression associated with reduced synchrony between metabolic gene expression and the diurnal rhythm of food intake [45]. Phase changes in insulin-dependent genes in SCAT biopsies, were suggested to contribute to increased daytime lipolysis in individuals with T2D [45]. Also, partial alteration of the circadian clock in human SCAT by sleep deprivation was associated with increased carbohydrate turnover and impaired glucose homeostasis [46]. On the other hand, weight loss of at least 8% over an 8-week diet in humans with obesity was associated with induction of circadian repressors in SCAT [47], similar to the acute effects of one exercise bout in our study. Thus, in line with this common notion that obesity reduces or alters the circadian rhythm in SCAT likely by reduction of amplitude, we report that human SCAT responds directly to acute exercise and training with an altered circadian expression pattern. Based on sampling time and peak time data we provide evidence that suggest an increased amplitude in SCAT as response to exercise in people with obesity. In addition to the observed potential restoration of a healthier circadian rhythm along with metabolic benefits as indicated by previous studies [45, 47] we observed acute and long-term adaptations of genes and enzymes associated with insulin signaling, lipid metabolism and inflammation, reported to be closely intertwined with the circadian rhythm. The improved metabolic control associated with a restored circadian rhythm could be one piece in the puzzle of how the adaptation of SCAT to acute exercise and training contributes to the systemic improvements observed after regularly performed exercise.

One controversially discussed topic of SCAT adaptation to exercise is mitochondrial respiration capacity and beigeing/browning. It is known that in SM mitochondrial respiration is induced in response to training [12, 23, 48] which we confirmed in our study accompanied by the strong acute induction of mitochondrial marker *PGC1α* expression in SM as an acute response to exercise bouts. This is in stark contrast to what we found in SCAT where neither *PGC1α* nor mitochondrial respiration was affected by the 8-week intervention. Rodent studies often indicate that adipocyte beigeing/browning may be an adaptation to regularly performed exercise, which increases the oxidative and thermogenic potential of the SCAT and may thereby contribute to the anti-diabetic actions of exercise training [49–51]. Human SCAT might respond to exercise or training with an altered OXPHOS expression profile however many studies fail to reproduce results observed in mice regarding beigeing/browning [21, 24, 52]. Our results fall right in line with these previous reports showing no changes in mitochondrial respiration capacity or browning genes, after the training intervention. It should be considered, however, that the induction of browning with exercise in rodents might also be an artifact of housing animals under sub-thermoneutral conditions which is the case in many laboratory setups. When mice are housed at thermoneutrality, the browning of SCAT appears to be absent also in exercising rodents, closely resembling what is seen in humans [53, 54].

Unlike many previously mentioned studies, our study design provided us with the unique opportunity to directly compare acute and long-term responses of SCAT to exercise with changes in the transcriptome of SMs of the same donors. The transcriptomic changes in muscle in response to acute exercise bouts in our study are well in line with the literature with *NR4A3* as one of the most exercise-responsive genes [13] and activation of amongst others PGC1α, ESSRA, VEGF, and STAT3-dependent gene expression [7]. What was most interesting, however, was the virtual lack of overlap between molecular adaptations to exercise/training between SM and SCAT in the same donors. The only two transcripts that were regulated in the same direction in response to acute exercise in both the trained and untrained state in the two tissues were *PDK4* (up) and *MYLIP* (down). PDK4 regulates a central switching point in glucose and fatty-acid metabolism by inhibition of pyruvate oxidation. The robust reduction in *MYLIP* in both SM and SCAT in response to acute exercise is reported in here for the first time. MYLIP is a E3 ubiquitin ligase that targets members of the LDLR family for degradation, with yet to be defined function in peripheral tissues [55]. As specific kinetics of individual transcripts in response to acute bouts of exercise in SCAT are unknown, a potential delay of response within the not directly utilized SCAT, might in part explain the lack of overlap in the transcriptomic responses which cannot be determined within an individual biopsy taken 60 min after exercise.

An intriguing question is the nature of the mediators of the acute transcriptional response in SCAT. While SM as physically utilized tissue during exercise is exposed to tremendous changes in blood flow, ATP consumption and mechanical stress, the stimuli for acute molecular responses in SCAT are less obvious. As insulin and cAMP are generally regarded to have opposing effects [56], our upstream-regulator analysis points to a contribution of activated cAMP-dependent signaling. This is most likely activated via β-adrenergic receptors stimulated by the exercise-dependent increase in catecholamines, which is also responsible for fatty acid mobilization through lipolysis thereby counteracting inhibition of lipolysis by insulin [32]. This is well in line with the observed inhibition of insulin-dependent upstream signaling, and activated β-adrenergic receptor signaling. Thus, β-adrenergic signaling as likely transmitter of exercise response to SCAT is supported by our upstream-regulator analysis based on the transcriptomic changes.

An inherent limitation of the presented study is the focus on transcriptomic data without validation on protein level. As we set out to characterize transcriptomic responses in two distinct exercise-responsive tissues of the same human donors and found not only long-term changes after 8 weeks of intervention but consistent regulation of individual transcripts to repeated bouts of exercise, a functional effect translating to the protein level can be assumed. Without validation on proteomic level in a similar untargeted matched approach, however, an inherent uncertainty remains that could be resolved by future studies. To minimize the temporal involvement and the physical load for the study participants, we were limited by the number of acceptable biopsies and the timepoints possible to collect biopsies. We chose 5 days after the last training bout for analyzing long-term effects on SCAT and SM, which might be too late to see pronounced training effects on transcript level. No additional resting biopsies were taken at the same day immediately before exercise. Also, the number of subjects and samples that could be included in this study, amounting to a total of *n* = 14 with *n* = 6 individual participants being represented in each timepoint, has to be kept in mind when considering the presented results. Further, a parallel control group without training would be desirable in future studies to compare the long-term effect in the trained group with an untrained group. Finally, our data were obtained in individuals with overweight or obesity. While body composition does not seem to impact anabolic response to exercise and muscle protein synthesis [57], it was previously suggested to have individual effects on training-induced compositional and functional changes of the human gut microbiota [58], amplitude of non-adrenergically and adrenergically mediated lypolysis [59] as well as timely onset of lipolysis post exercise in adipose tissue [60]. Thus, observed responses in our cohort likely differ from results expected in lean individuals regarding adipose tissue adaptation which makes such a control desirable as well in future studies. This study however, solely focused on individuals that would benefit most from preventative effects of exercise regarding onset of metabolic syndrome and T2D.

## Conclusion

Adipose tissue responds completely distinct from adaptations of skeletal muscle with acute and repeated reduction in transcripts related to lipid uptake, synthesis and storage. The results suggest a potential restoration of a healthier circadian rhythm in adipose tissue of subjects with obesity after regularly performed exercise. The interconnection of circadian rhythm with lipid metabolism possibly contributes to improved metabolic health and prevention of type 2 diabetes. Our findings on lipid metabolism and circadian rhythm as well as on potential mediators of adipose tissue exercise response are intended to spark future research with the overall goal to fully understand and characterize the positive effect of exercise on diabetes prevention on a molecular level.

## Supplementary information

Supplementary Methods

Supplementary Data

Supplementary Figure 1

Supplementary Figure 2

Supplementary Table 1

Supplementary Table 2

## Data Availability

The transcriptomic data used in this study is available via the GEO database at NCBI (GSE224310). Further data that support the findings of this study are available from the corresponding author upon request.
