# Supplementary material for: Acute and long-term exercise adaptation of adipose tissue and skeletal muscle in humans: a matched transcriptomics approach after 8-week training-intervention
Source: Int J Obes (Lond). 2023 Feb 11;47(4):313–24. doi: 10.1038/s41366-023-01271-y (PMC10113153; doi:10.1038/s41366-023-01271-y)
Supplement: Supplementary file 1 — Supplementary Methods [file 41366_2023_1271_MOESM1_ESM.docx]

# Supplementary Methods

## High-Resolution Respirometry

Respirometry measurements were performed using Oroboros Oxygraph 2k (Oroboros Instruments GmbH, Innsbruck, Austria) as described previously in (26). Data of the subset (n=14) of all participants were included in this study. In brief, muscle tissue single fibers were prepared and permeabilized in saponin solution (50μg/mL in MIR05) at 4 °C. For measurement, 2mg of permeabilized and washed fibers were placed in the measurement chamber. From SCAT, roughly 50mg tissue were minced in 700μL MIR05 on ice. The minced tissue was then transferred to the measurement chamber and permeabilized using digitonin solution in a final concentration of 12μM. Malate (1.28mM), octanoylcarnitine (0.5mM), adenosine diphosphate (ADP; 2.5mM), pyruvate (5mM), succinate (2.5mM), and cytochrome c (10μM) were added. FCCP was titrated in 125nM steps for muscle and 1μM steps for SCAT. Finally, rotenone (1.25μM) and antimycin A (5μM) were added. Non-mitochondrial oxygen consumption (after antimycin injection) was subtracted from all flux rates. Flux rates were normalized to tissue wet weight.

## Adipose tissue transcriptomic analysis in the CLOCK study

In the CLOCK study (NCT02487576), from 15 out of 29 non-diabetic men (age 45.9 ± 2.5 years, BMI 27.1 ± 0.8 kg/m2, fasting venous glucose levels < 126 mg/dL and 2h glucose levels < 200 mg/dL in the 75-g OGTT), transcriptomic analysis of SCAT samples was conducted (30). Exclusion criteria were weight changes > 2 kg within past 2 months, current shift work or history of shift work and diseases or conditions that might influence the circadian rhythms or metabolic outcomes. SCAT biopsies were collected three times during the investigation day (at 8:40 am, 12:20 pm and 7:00 pm) at the level of the umbilicus by the needle aspiration. Before the investigation day, subjects consumed a diet consisted of a high-carb breakfast and lunch and a high-fat snack and dinner for four weeks as described (30). Total RNA was purified using the miRNeasy Lipid Tissue Mini Kit (Qiagen, Germany), and RNA quality was assessed by Agilent 2100 bioanalyzer using Agilent RNA 6000 Nano Kit (Agilent Technologies, Germany). Biotinylated cRNA were prepared according to the standard Affymetrix protocol and hybridized to GeneChip Human Gene 2.0 ST Arrays (Affymetrix, Germany) for 16h at 45° C. Microarrays were scanned with an Affymetrix GeneArray Scanner 3000. CustomCDF Version 14 with Entrez based gene definitions was used for gene annotation. Parameters of circadian rhythms of gene expression were estimated by a three-time-point rhythm prediction method (31) using a magnitude correction.

## Statistical analyses

Anthropometric data and linear regression models were analyzed with R3.6.3/RStudio. Normality was tested by Shapiro-Wilk-test from the R package ‘stats’ (v3.6.3) and non-normal data were log-transformed. Genewise testing for differential gene expression between time points were calculated employing the (paired) limma t-test using the R package ‘limma’ (v3.46.0). To reduce background, gene sets were filtered using detection above background p-values <0.05 in more than 50% of the samples in at least one of the treatment groups per comparison. For correction of multiple testing p-values were adjusted with Benjamini-Hochberg FDR<10% (BH). Data were given as mean±SD and p-values <0.05 were considered significant. Differences between all timepoints were assessed using one-way ANOVA with Tukey correction for multiple comparisons. Graphs were made using the R packages ‘ggplot2’ (v3.3.2) and ‘ggrepel’ (v0.9.1) and figures assembled using InkScape (v1.0). Functional enrichment analysis was carried out online using <https://biit.cs.ut.ee/gprofiler/gost> with p<0.05 as threshold and g:SCS as method for multiple testing correction. Homo sapiens was chosen as organism and analysis was performed using the databases from KEGG, Wikipathways (WP) and Reactome (REAC). For upstream regulator analysis we used QIAGEN’s Ingenuity Pathway Analysis software (IPA®, QIAGEN Redwood City, content v70750971) with the reference set to the Ingenuity Knowledge Base (genes only) including direct and indirect relationships.
