# Supplementary material for: Acute and long-term exercise adaptation of adipose tissue and skeletal muscle in humans: a matched transcriptomics approach after 8-week training-intervention
Source: Int J Obes (Lond). 2023 Feb 11;47(4):313–24. doi: 10.1038/s41366-023-01271-y (PMC10113153; doi:10.1038/s41366-023-01271-y)
Supplement: Supplementary file 2 — Supplementary Data [file 41366_2023_1271_MOESM2_ESM.docx]

# Supplementary Data


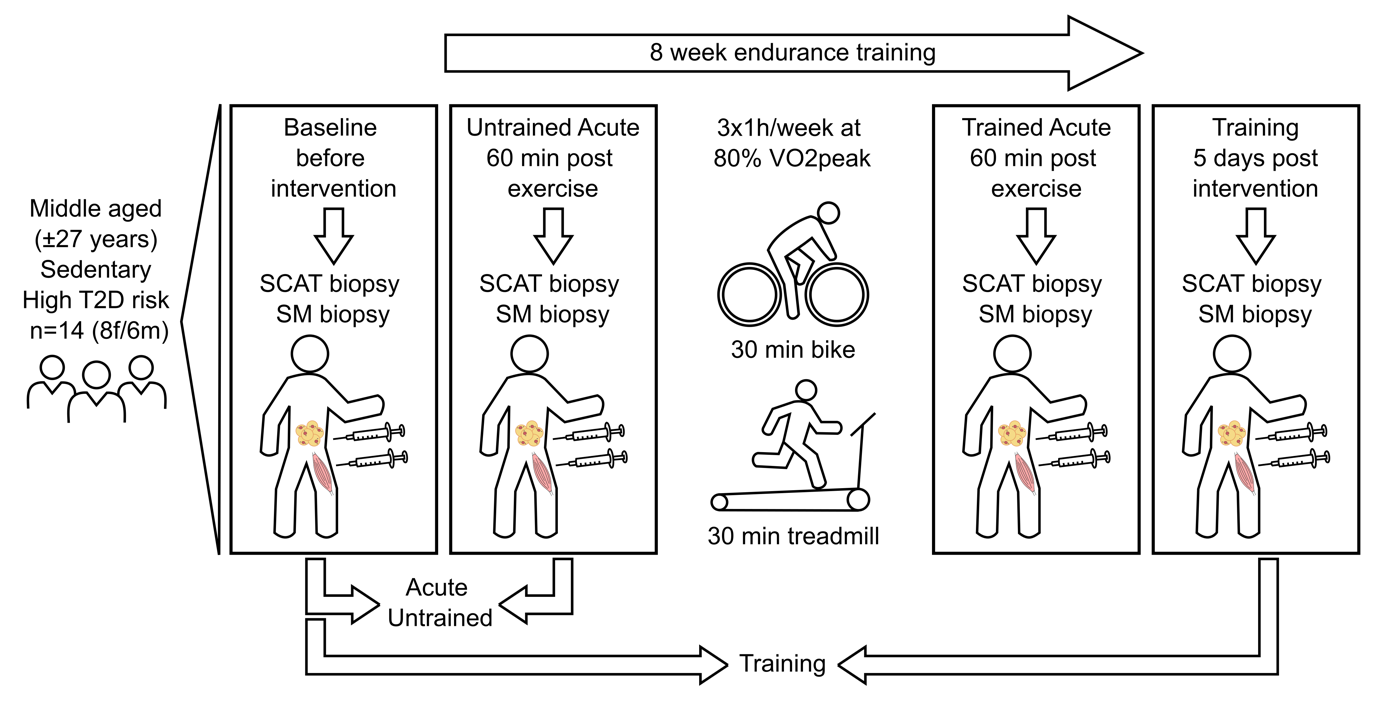


Fig. S 1 Study design. Healthy, sedentary humans (27±4 yrs) at high risk for T2D (n=14, 8 female / 6 male) performed one hour of supervised endurance training three times per week for 8 weeks, consisting of 30 minutes of cycling and 30 minutes of walking on a treadmill. Individual training intensity was set at 80% of VO_2_peak and kept constant. Subcutaneous adipose tissue biopsies (SCAT) and skeletal muscle (SM) biopsies were taken before (Baseline) and 5 days after (Trained) the 8-week intervention in a resting state 60 min after the end of an OGTT as well as 60 min after the first (Untrained Acute) and last 30 min ergometer exercise bout (Trained Acute). At the acute dates, participants received a defined breakfast to account for the OGTT-induced hormonal changes at the resting state. All biopsies were collected at 11:00 am ± 30 minutes.


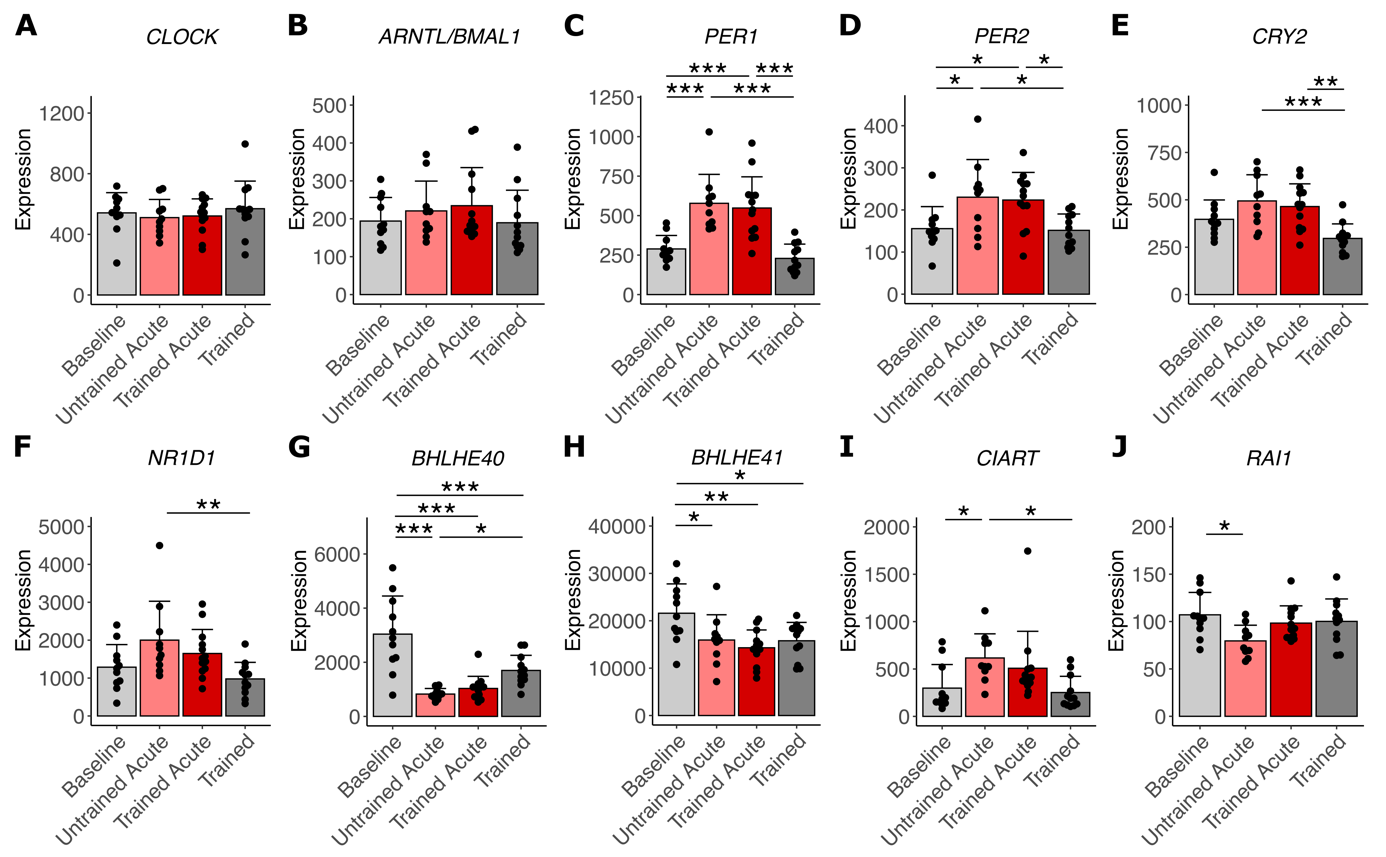


Fig. S 2 Circadian gene expression during acute exercise and training. Subcutaneous adipose tissue biopsies of participants that underwent an 8-week training intervention program were analyzed. All biopsies were taken at 11:00 am ± 30 minutes, before (Baseline n=11) and after the intervention (Trained n=12) as well as 60 minutes after the first (Untrained Acute n=10) and last acute exercise bout (Trained Acute n=13). Transcript levels of A) *CLOCK*, B) *ARNTL/BMAL1*, C) *PER1*, D) *PER2*, E) *CRY2*, F) *NR1D1*, G) *BHLHE40*, H) *BHLHE41*, I) *CIART*, J) *RAI1* were compared between each timepoint. Bars represent mean±SD, individual datapoints are depicted. Significant differences were assessed using one-way ANOVA with Tukey correction, * p<0.05, ** p<0.01, *** p<0.001, n=10-13 with n=6 represented in all timepoints.

Tab. S 1 Study participants

| Parameter | Pre | Post-8-week | p-Value |
| --- | --- | --- | --- |
| **Sex** | 8 female / 6 male | | |
| **Age [years]** |  | 27.90 ± 4.11  (19.0 – 35.0) |  |
| **Height [cm]** | 171 ± 9.47  (157 – 187) | 171 ± 9.47  (157 – 187) |  |
| **Body mass [kg]** | 92.1 ± 15.7  (71.9 – 132) | 91.7 ± 15.7  (73.0 – 130) | 0.574 |
| **BMI [kg/m²]** | 31.20 ± 3.67  (27.5 – 40.0) | 31.10 ± 3.90  (26.3 – 39.5) | 0.662 |
| **Waist to hip ratio** | 0.89 ± 0.04  (0.81 – 0.96) | 0.88 ± 0.05  (0.79 – 0.97) | 0.466 |
| **Total AT volume [l]** | 39.30 ± 10.10  (25.3 – 58.2) | 38.50 ± 9.95  (22.8 – 56.7) | 0.064 |
| **Subcutaneous AT [l]** | 14.90 ± 5.00  (8.42 – 24.60) | 14.30 ± 4.85  (7.20 – 23.40) | 0.053 |
| **Visceral AT [l]** | 3.01 ± 1.11  (1.40 – 5.42) | 2.88 ± 1.03  (1.39 – 5.23) | 0.160 |
| **IATergo/BM [W/kg]** | 1.09 ± 0.19  (0.77 – 1.44) | 1.39 ± 0.26  (0.96 – 1.87) | <0.001 |
| **VO2peak/BM [ml/(kg*min)]** | 24.80 ± 3.35  (18.7 – 29.2) | 27.90 ± 5.14  (16.0 – 34.9) | 0.004 |
| **Glucose fasting [mmol/l]** | 5.14 ± 0.32  (4.61 – 5.72) | 5.10 ± 0.28  (4.61 – 5.61) | 0.563 |
| **Glucose OGTT_120 min_ [mmol/l]** | 5.51 ± 0.94  (4.22 – 7.50) | 5.55 ± 1.87  (4.00 – 11.60) | 0.077 |
| **Insulin fasting [pmol/l]** | 92.1 ± 32.0  (45.0 – 150) | 93.5 ± 29.4  (50.0 – 135) | 0.824 |
| **Insulin OGTT_120 min_ [pmol/l]** | 511 ± 385  (65 – 1539) | 439 ± 365  (61 – 1345) | 0.069 |
| **ISIMats** | 9.73 ± 5.75  (3.89 – 27.00) | 10.00 ± 4.98  (4.47 – 21.40) | 0.696 |
| **HbA1c [mmol/mol Hb]** | 34.00 ± 2.07  (31.0 – 39.0) | 33.50 ± 1.76  (30.0 – 36.0) | 0.764 |
| **HbA1c [%]** | 5.26 ± 0.19  (4.99 – 5.72) | 5.22 ± 0.16  (4.89 – 5.44) | 0.764 |

AT: adipose tissue, BP: blood pressure, IAT: individual aerobic threshold, BM: body mass, ergo: bicycle ergometer ISI: insulin sensitivity index. Paired t-tests or Wilcoxon signed rank tests when data were not normally distributed. N = 14, mean ± SD, results are subset of recently published data (26).

Tab. S 2 Circadian Peak Times in adipose tissue from the CLOCK study (NCT03151590)

|  | peak time | Cosinor fitting | | |
| --- | --- | --- | --- | --- |
|  |  | p-Value | r.squared | fdr |
| *AACS* | 20:04 | 0.001 | 0.281 | 0.004 |
| *ACACA* | no |  |  |  |
| *ACLY* | 18:54 | 0.001 | 0.290 | 0.003 |
| *ADA* | 16:15 | 0.002 | 0.260 | 0.006 |
| *ADORA1* | no |  |  |  |
| *ANGPTL4* | no |  |  |  |
| *ANGPTL8* | no |  |  |  |
| *ARNTL/BMAL1* | 20:07 | <0.001 | 0.393 | <0.001 |
| *BHLHE40* | 14:27 | 0.035 | 0.147 | 0.051 |
| *BHLHE41* | no |  |  |  |
| *CHD9* | no |  |  |  |
| *CIART* | 10:09 | <0.001 | 0.522 | <0.001 |
| *CLOCK* | 23:05 | 0.001 | 0.300 | 0.003 |
| *CRTC3* | no |  |  |  |
| *CRY2* | 11:56 | 0.016 | 0.179 | 0.027 |
| *DRD1* | 14:31 | 0.016 | 0.179 | 0.028 |
| *ELOVL6* | 22:36 | 0.003 | 0.247 | 0.008 |
| *FASN* | no |  |  |  |
| *GPAM* | no |  |  |  |
| *HCRTR2* | no |  |  |  |
| *HTR7* | 01:29 | 0.005 | 0.223 | 0.012 |
| *ID2* | no |  |  |  |
| *INSIG1* | 20:34 | 0.003 | 0.237 | 0.009 |
| *IRS1* | 19:44 | <0.001 | 0.515 | <0.001 |
| *JUN* | no |  |  |  |
| *KLF9* | 08:09 | <0.001 | 0.740 | <0.001 |
| *KLF10* | 13:37 | 0.006 | 0.214 | 0.014 |
| *LDLR* | 20:28 | 0.092 | 0.107 | 0.111 |
| *MID1IP1* | 15:47 | 0.012 | 0.189 | 0.023 |
| *NFIL3* | 18:12 | <0.001 | 0.659 | <0.001 |
| *NLGN1* | no |  |  |  |
| *NR1D1* | 06:57 | <0.001 | 0.704 | <0.001 |
| *PCK1* | 20:49 | <0.001 | 0.386 | <0.001 |
| *PDK4* | 03:44 | <0.001 | 0.489 | <0.001 |
| *PER1* | 08:32 | <0.001 | 0.747 | <0.001 |
| *PER2* | 11:03 | <0.001 | 0.466 | <0.001 |
| *PLIN2* | no |  |  |  |
| *PNPLA3* | 18:31 | 0.081 | 0.113 | 0.100 |
| *PPARG* | no |  |  |  |
| *PTGDS* | no |  |  |  |
| *RAI1* | 18:18 | <0.001 | 0.431 | <0.001 |
| *RORB* | 19:47 | 0.018 | 0.175 | 0.030 |
| *RXRA* | no |  |  |  |
| *SERPINE1* | no |  |  |  |
| *SIN3A* | no |  |  |  |
| *SREBF1* | 17:25 | <0.001 | 0.374 | 0.001 |
| *XBP1* | 16:52 | <0.001 | 0.494 | <0.001 |

Peak time: Time of day with maximum expression based on a cosinor fitting of 3 sample datapoints of one day as described in Pivovarova, Jurchott et al. 2015 from samples assessed in Kessler, Hornemann et al. 2018; p-Value: based on fitting of the 3 sample datapoints of one day on the cosinor-curve p<0.05 suggests a circadian expression pattern; r.squared: r2 value for cosinor fitting; fdr: p-value after correction for multiple testing, p<0.05 is a circadian expression pattern; no: no cosinor fitting possible based on 3 sample datapoints of one day, no circadian expression pattern
