# Supplementary figures and images for: Acute and long-term exercise adaptation of adipose tissue and skeletal muscle in humans: a matched transcriptomics approach after 8-week training-intervention
Source: Int J Obes (Lond). 2023 Feb 11;47(4):313–24. doi: 10.1038/s41366-023-01271-y (PMC10113153; doi:10.1038/s41366-023-01271-y)

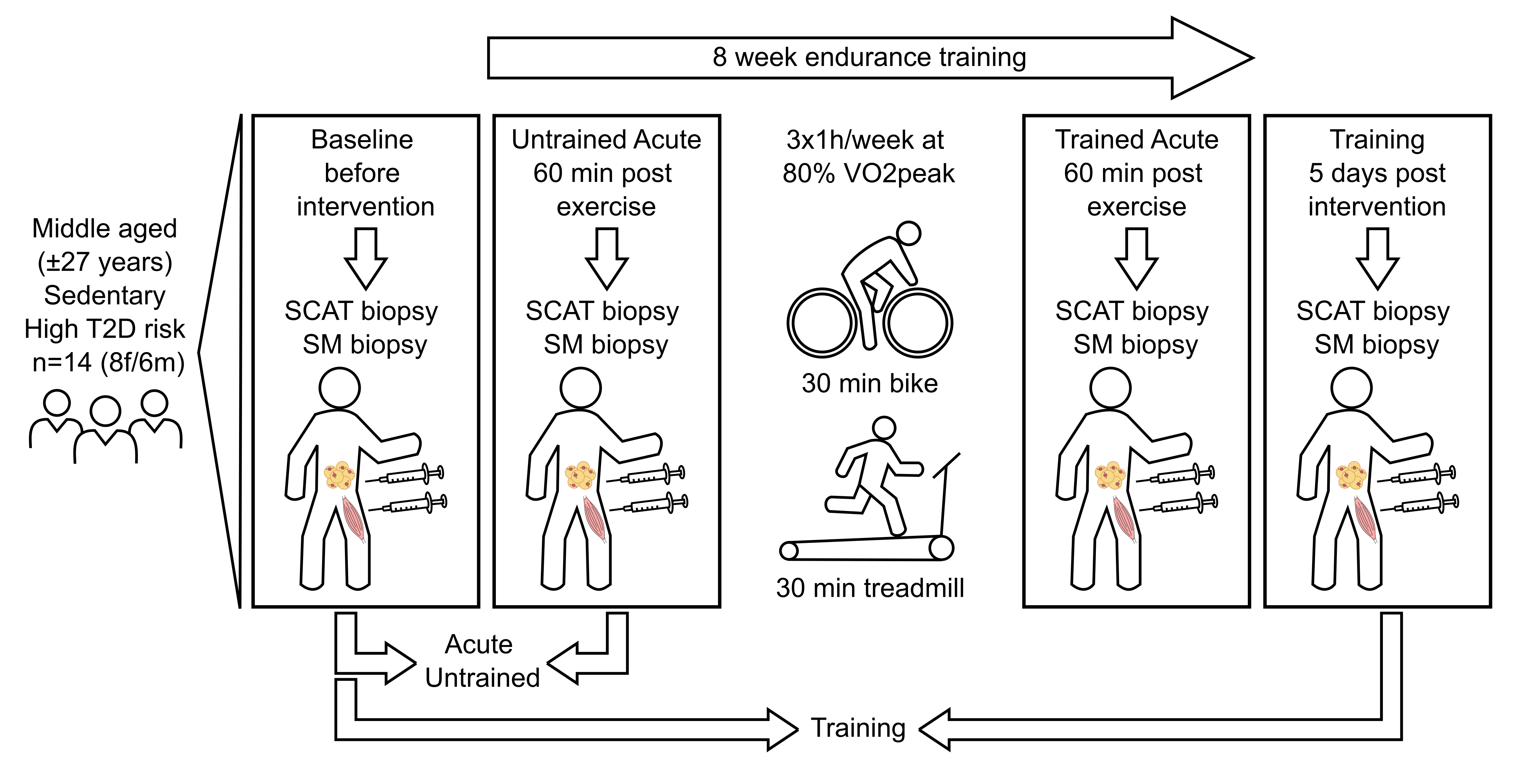

Supplement: Supplementary file 3 — Supplementary Figure 1 [file 41366_2023_1271_MOESM3_ESM.png]

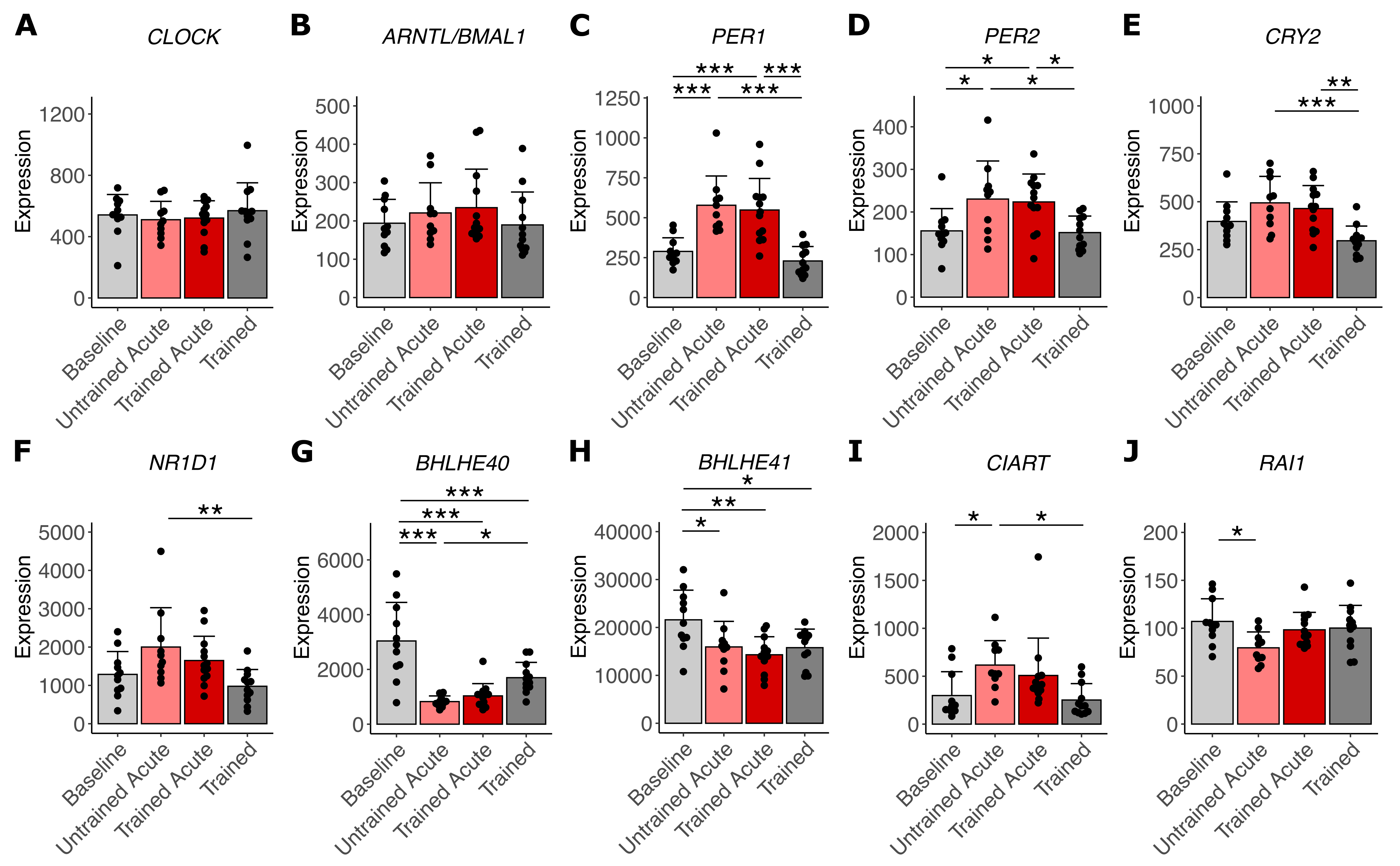

Supplement: Supplementary file 4 — Supplementary Figure 2 [file 41366_2023_1271_MOESM4_ESM.png]
