# Supplementary material for: Acute and long-term exercise adaptation of adipose tissue and skeletal muscle in humans: a matched transcriptomics approach after 8-week training-intervention
Source: Int J Obes (Lond). 2023 Feb 11;47(4):313–24. doi: 10.1038/s41366-023-01271-y (PMC10113153; doi:10.1038/s41366-023-01271-y)
Supplement: Supplementary file 5 — Supplementary Table 1 [file 41366_2023_1271_MOESM5_ESM.docx]

Tab. S 1 Study participants

| Parameter | Pre | Post-8-week | p-Value |
| --- | --- | --- | --- |
| **Sex** | 8 female / 6 male | | |
| **Age [years]** |  | 27.90 ± 4.11  (19.0 – 35.0) |  |
| **Height [cm]** | 171 ± 9.47  (157 – 187) | 171 ± 9.47  (157 – 187) |  |
| **Body mass [kg]** | 92.1 ± 15.7  (71.9 – 132) | 91.7 ± 15.7  (73.0 – 130) | 0.574 |
| **BMI [kg/m²]** | 31.20 ± 3.67  (27.5 – 40.0) | 31.10 ± 3.90  (26.3 – 39.5) | 0.662 |
| **Waist to hip ratio** | 0.89 ± 0.04  (0.81 – 0.96) | 0.88 ± 0.05  (0.79 – 0.97) | 0.466 |
| **Total AT volume [l]** | 39.30 ± 10.10  (25.3 – 58.2) | 38.50 ± 9.95  (22.8 – 56.7) | 0.064 |
| **Subcutaneous AT [l]** | 14.90 ± 5.00  (8.42 – 24.60) | 14.30 ± 4.85  (7.20 – 23.40) | 0.053 |
| **Visceral AT [l]** | 3.01 ± 1.11  (1.40 – 5.42) | 2.88 ± 1.03  (1.39 – 5.23) | 0.160 |
| **IATergo/BM [W/kg]** | 1.09 ± 0.19  (0.77 – 1.44) | 1.39 ± 0.26  (0.96 – 1.87) | <0.001 |
| **VO2peak/BM [ml/(kg*min)]** | 24.80 ± 3.35  (18.7 – 29.2) | 27.90 ± 5.14  (16.0 – 34.9) | 0.004 |
| **Glucose fasting [mmol/l]** | 5.14 ± 0.32  (4.61 – 5.72) | 5.10 ± 0.28  (4.61 – 5.61) | 0.563 |
| **Glucose OGTT_120 min_ [mmol/l]** | 5.51 ± 0.94  (4.22 – 7.50) | 5.55 ± 1.87  (4.00 – 11.60) | 0.077 |
| **Insulin fasting [pmol/l]** | 92.1 ± 32.0  (45.0 – 150) | 93.5 ± 29.4  (50.0 – 135) | 0.824 |
| **Insulin OGTT_120 min_ [pmol/l]** | 511 ± 385  (65 – 1539) | 439 ± 365  (61 – 1345) | 0.069 |
| **ISIMats** | 9.73 ± 5.75  (3.89 – 27.00) | 10.00 ± 4.98  (4.47 – 21.40) | 0.696 |
| **HbA1c [mmol/mol Hb]** | 34.00 ± 2.07  (31.0 – 39.0) | 33.50 ± 1.76  (30.0 – 36.0) | 0.764 |
| **HbA1c [%]** | 5.26 ± 0.19  (4.99 – 5.72) | 5.22 ± 0.16  (4.89 – 5.44) | 0.764 |

AT: adipose tissue, BP: blood pressure, IAT: individual aerobic threshold, BM: body mass, ergo: bicycle ergometer ISI: insulin sensitivity index. Paired t-tests or Wilcoxon signed rank tests when data were not normally distributed. N = 14, mean ± SD, results are subset of recently published data (26).
