# Supplementary material for: Acute and long-term exercise adaptation of adipose tissue and skeletal muscle in humans: a matched transcriptomics approach after 8-week training-intervention
Source: Int J Obes (Lond). 2023 Feb 11;47(4):313–24. doi: 10.1038/s41366-023-01271-y (PMC10113153; doi:10.1038/s41366-023-01271-y)
Supplement: Supplementary file 6 — Supplementary Table 2 [file 41366_2023_1271_MOESM6_ESM.docx]

Tab. S 2 Circadian Peak Times in adipose tissue from the CLOCK study (NCT03151590)

|  | peak time | Cosinor fitting | | |
| --- | --- | --- | --- | --- |
|  |  | p-Value | r.squared | fdr |
| *AACS* | 20:04 | 0.001 | 0.281 | 0.004 |
| *ACACA* | no |  |  |  |
| *ACLY* | 18:54 | 0.001 | 0.290 | 0.003 |
| *ADA* | 16:15 | 0.002 | 0.260 | 0.006 |
| *ADORA1* | no |  |  |  |
| *ANGPTL4* | no |  |  |  |
| *ANGPTL8* | no |  |  |  |
| *ARNTL/BMAL1* | 20:07 | <0.001 | 0.393 | <0.001 |
| *BHLHE40* | 14:27 | 0.035 | 0.147 | 0.051 |
| *BHLHE41* | no |  |  |  |
| *CHD9* | no |  |  |  |
| *CIART* | 10:09 | <0.001 | 0.522 | <0.001 |
| *CLOCK* | 23:05 | 0.001 | 0.300 | 0.003 |
| *CRTC3* | no |  |  |  |
| *CRY2* | 11:56 | 0.016 | 0.179 | 0.027 |
| *DRD1* | 14:31 | 0.016 | 0.179 | 0.028 |
| *ELOVL6* | 22:36 | 0.003 | 0.247 | 0.008 |
| *FASN* | no |  |  |  |
| *GPAM* | no |  |  |  |
| *HCRTR2* | no |  |  |  |
| *HTR7* | 01:29 | 0.005 | 0.223 | 0.012 |
| *ID2* | no |  |  |  |
| *INSIG1* | 20:34 | 0.003 | 0.237 | 0.009 |
| *IRS1* | 19:44 | <0.001 | 0.515 | <0.001 |
| *JUN* | no |  |  |  |
| *KLF9* | 08:09 | <0.001 | 0.740 | <0.001 |
| *KLF10* | 13:37 | 0.006 | 0.214 | 0.014 |
| *LDLR* | 20:28 | 0.092 | 0.107 | 0.111 |
| *MID1IP1* | 15:47 | 0.012 | 0.189 | 0.023 |
| *NFIL3* | 18:12 | <0.001 | 0.659 | <0.001 |
| *NLGN1* | no |  |  |  |
| *NR1D1* | 06:57 | <0.001 | 0.704 | <0.001 |
| *PCK1* | 20:49 | <0.001 | 0.386 | <0.001 |
| *PDK4* | 03:44 | <0.001 | 0.489 | <0.001 |
| *PER1* | 08:32 | <0.001 | 0.747 | <0.001 |
| *PER2* | 11:03 | <0.001 | 0.466 | <0.001 |
| *PLIN2* | no |  |  |  |
| *PNPLA3* | 18:31 | 0.081 | 0.113 | 0.100 |
| *PPARG* | no |  |  |  |
| *PTGDS* | no |  |  |  |
| *RAI1* | 18:18 | <0.001 | 0.431 | <0.001 |
| *RORB* | 19:47 | 0.018 | 0.175 | 0.030 |
| *RXRA* | no |  |  |  |
| *SERPINE1* | no |  |  |  |
| *SIN3A* | no |  |  |  |
| *SREBF1* | 17:25 | <0.001 | 0.374 | 0.001 |
| *XBP1* | 16:52 | <0.001 | 0.494 | <0.001 |

Peak time: Time of day with maximum expression based on a cosinor fitting of 3 sample datapoints of one day as described in Pivovarova, Jurchott et al. 2015 from samples assessed in Kessler, Hornemann et al. 2018; p-Value: based on fitting of the 3 sample datapoints of one day on the cosinor-curve p<0.05 suggests a circadian expression pattern; r.squared: r2 value for cosinor fitting; fdr: p-value after correction for multiple testing, p<0.05 is a circadian expression pattern; no: no cosinor fitting possible based on 3 sample datapoints of one day, no circadian expression pattern
